# Ultrasound-Guided Lateral Pectoral Nerve Pulsed Radiofrequency in a Patient With Refractory Pectoral Pain: A Case Report of a Novel Approach

**DOI:** 10.7759/cureus.21680

**Published:** 2022-01-28

**Authors:** Inês Ferro, Adriana Pereira, Ana F Gonçalves, Joana Martins, José L Carvalho

**Affiliations:** 1 Physical Medicine and Rehabilitation, Centro de Medicina de Reabilitação da Região Centro - Rovisco Pais, Tocha, PRT; 2 Physical Medicine and Rehabilitation, Centro de Reabilitação de Alcoitão, Alcoitão, PRT; 3 Physical Medicine and Rehabilitation, Centro de Reabilitação do Norte, Gaia, PRT; 4 Physical Medicine and Rehabilitation, Centro Hospitalar e Universitário de Coimbra, Coimbra, PRT

**Keywords:** pain management, pulsed radiofrequency, ultrasound injection, pectoral pain, lateral pectoral nerve injury

## Abstract

The lateral pectoral nerve is often injured along with the brachial plexus, but its isolated lesions are rare. We report a clinical case of an isolated lateral pectoral nerve injury, presenting as a refractory right shoulder and pectoral pain, determining functional repercussion. After clinical assessment and imaging investigation, it was considered that the pain source was likely to be a lateral pectoral nerve mononeuropathy. Thus, a diagnostic ultrasound-guided nerve block was performed, with a major improvement in the patient's symptoms and functionality for two months. Thereafter, a long-lasting alternative was proposed - pulsed radiofrequency. As a form of neuromodulation, pulsed radiofrequency offers pain control without tissue damage or painful sequelae, which is usually associated with conventional radiofrequency.

## Introduction

The lateral pectoral nerve (LPN) is commonly injured along with the brachial plexus, but its isolated lesions are scarce. There are only a few reports in the literature regarding isolated LPN injury, related to sports, compression by the use of seat belts or repeated contractions of the pectoralis muscle related to training regimens, or iatrogenic to surgical procedures such as breast augmentation surgery [1-3].

Clinical findings of lateral pectoral nerve injury may include asymmetry of the chest wall associated with atrophy and weakness of the entire pectoralis major muscle or parts of it, and sometimes there is also pain associated. This might be explained by the fact that although this nerve mostly carries motor fiber, it is also considered to carry proprioceptive and nociceptive fibers, responsible for a part of the anterior glenohumeral joint complex innervation [2-3]. Plus, a partially denervated muscle or, after a while, an atrophic and weak pectoralis major muscle, when solicited, may conduct to spasms, which are described as producing intense pain.

Non-ablative pulsed radiofrequency (pRF) neuromodulation is an interventional pain management method. In contrast with continuous radiofrequency, pRF offers the advantage of pain control without tissue destruction or painful sequelae. This is especially alluring in neuropathic pain [4].

In this case report, we present a case of isolated damage to the LPN, without evident motor manifestation but with intense pain in the major pectoral muscle region.

## Case presentation

We describe a case of a 65-year-old female who works as a cleaning lady. She came to the outpatient department of physical rehabilitation medicine complaining of right shoulder pain, with consequent functional limitation. Her complaints started about one year ago, with no history of trauma, fall, or any other apparent injury. It was characterized as a mechanical pain, with a paroxistic irradiation to her upper ipsilateral chest area and proximal medial brachial region, which worsened with the arm movements and weight lifting that was an integral part of her work. She scored a maximum pain intensity of 8/10 on the Visual Numeric Scale (VNS). There were no nocturnal complaints, no weakness reported, no red flags. There was an insignificant improvement with analgesics, non-steroidal anti-inflammatory drugs (NSAIDs), and physical therapy.

The functional limitation consisted on the inability to perform some daily living activities but mostly her usual labor chores, because of the intense pain it elicited. The use of several repetitive upper limb movements, predominantly on her right arm (as this is her dominant side), as well as lifting and carrying of different weights, were the major movements that highly intensified her pain.

Upon observation, there was no limitation in active shoulder or cervical range-of-motion and no pain. There was no visible muscle wasting or asymmetries on her pectoral region. Shoulder special tests (including impingement, rotator cuff, deltoid and bicipital integrity) were all negative, but the Spurling test mimicked somewhat her usual right irradiated pain. There was a slight decrease in normal muscle force in the right C7 key muscle (comparing to her left side), without sensory alterations in any territory. There were no other alterations worth mentioning. There was tenderness on her pectoralis major region at palpation. An ultrasound was performed to her shoulder girdle, with no relevant findings.

As her physical examinations were inconclusive regarding her shoulder girdle, her pectoral region was apparently unremarkable as well, and with a suspicion of a C7 radiculopathy, a cervical MRI was requested. The cervical MRI showed merely posterior protrusions from C3 to C7, most expressive in C5-C6, slightly contacting the roots of C6, bilaterally.

**Figure 1:**
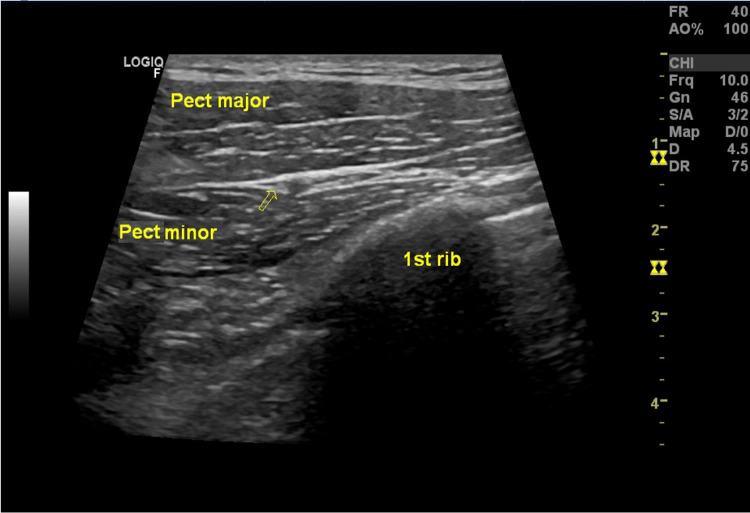
Ultrasound image of the lateral pectoral nerve (arrow), in the fascia between the pectoralis major muscle and pectoralis minor muscle. Pect major: pectoralis major muscle; Pect minor: pectoralis minor muscle

In the next appointment, about one month later, her pain pattern was still very expressive in her pectoral region and right shoulder. After another clinical examination, and bringing into account that there was no motor or sensory alterations present (compatible with C6 or C7 radiculopathy), we considered that the source of most pain was likely from an LPN neuropathy or entrapment. We proposed a diagnostic test with an ultrasound-guided nerve block with an anesthetic. For the procedure, the patient was positioned in supine, and with a 3.7-13 MHz linear probe (Logiq™ P8 ultrasound machine, GE Healthcare, Chicago, Illinois, United States), the LPN was identified in the fascial plane between the pectoralis major and minor muscles, next to the thoracoacromial artery (Figure 1). After the aseptic measures, a 22-gauge needle (0.8x40mm) was guided in a lateral to medial approach to our target and once the needle tip was in the interfascial plane, 5 ml of a mixture of 2% lidocaine and 0,2% ropivacaine was injected. The diagnostic test was positive, with a major improvement in pain (maximum VNS 0-1/10) and function that lasted for two months with minimum pain while working and performing activities with her right upper limb and without the need for analgesics.

**Figure 2:**
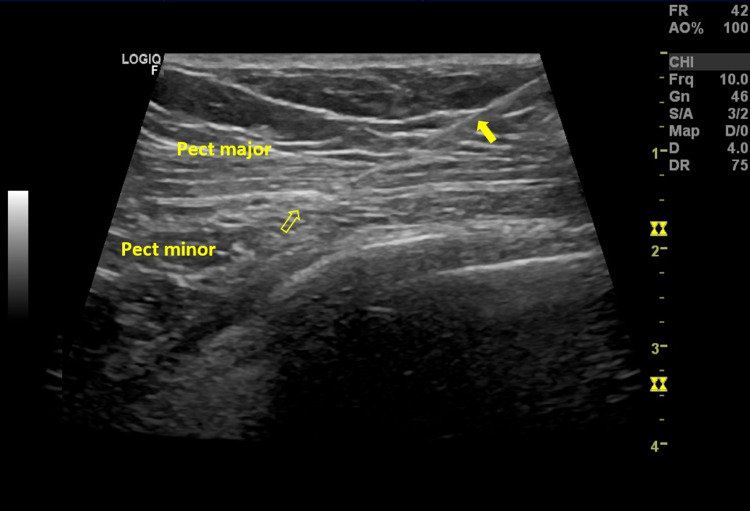
Ultrasound image of the needle tip (solid arrow) near the lateral pectoral nerve (arrow). Pect major: pectoralis major muscle; Pect minor: pectoralis minor muscle

After this, we proposed a more prolonged alternative - pulsed radiofrequency. The ultrasound-guided procedure was done with the same methodology previously described, using a specific 22-gauge radiofrequency cannula - 10cm x 10mm x 22ga (0,7mm) - with the tip near the nerve. A sensitive and motor test were also performed to additional confirmation. The pRF was conducted with the parameters: 100 V, frequency 2 Hz, 42ºC, with pulses of 2 ms, for 6 minutes. After the pRF cycle, 5 ml of 0,2% ropivacaine were administered in the same place, performing an hydrodissection of the fascia (Figure 2). There was an immediate response in pain after the local anesthetic administration, scoring her pain in VNS 1/10, even when performing movements that generated pain before. There were no complications after the procedure. A follow-up was schedule at three, six and twelve months, in order to perceive its medium and long-term benefits.

## Discussion

The LPN is a branch of the lateral cord of the brachial plexus. Occasionally, it also may arise from the anterior divisions of the upper and middle trunks of the brachial plexus. The LPN carries fibers from C5, C6, and C7 spinal nerves. After emerging deep to the clavicle, it runs anteriorly across the axillary artery and vein, pierces the clavipectoral fascia, and reaches the deep surface of pectoralis major muscles, splitting into four to seven branches. This nerve mostly carries motor fibers, but it has also been considered to carry proprioceptive and nociceptive fibers. It provides motor innervation primarily to the proximal two-thirds of the pectoralis major muscle, but recently some anatomical studies showed that there might be a sensitive innervation contributing to the anterior portion of the glenohumeral joint, subcoracoid region, and inferior portion of the acromioclavicular joint [5,6].

Anatomical studies of Gardetto et al. showed that the nerve branches of the LPN, having to pierce through a connective tissue septum that is thicker here by a few millimeters, may be subjected to additional risk of compression [7]. So, compression injury by repetitive muscle contractions may be of pathogenic relevance.

Since there was no report of any trauma to the region, we had to consider other possible causes for injury. Based on the anatomy of the LPN and medical history, there are some possible mechanisms for nerve damage. On one hand, intraneuronal axon damage may happen due to nerve compression by muscles nearby (such as the subclavian muscle) or where the nerve pierces the thick clavipectoral fascia, or where it encircles the muscle. On the other hand, a series of microtraumas during her occupational activities caused by intermittent but chronic load on her right arm and chest area could lead to nerve compression or stretch as it passes through the thick clavipectoral fascia, producing intraneural ischemia, edema, and bleeding, with consequent neuroma growth and nerve dysfunction.

Our patient's pain pattern was very expressive in her pectoral region and shoulder, probably related to spasms of the pectoralis major muscle during her activities, which usually result in severe pain (acute or chronic). As there were no motor or sensory alterations compatible with radiculopathy, we considered that the source of pain was likely to be an LPN mononeuropathy or entrapment. The ultrasound-guided LPN block, which had an essential role in the differential diagnosis of refractory shoulder pain, confirmed our theory. With that in line, a neuromodulation procedure with ultrasound-guided pRF was used to control these symptoms.

The increasing use of ultrasound to identify tissues and, particularly, fascial layers has led to the development of several interfascial injection techniques for analgesia. High-resolution ultrasonography is an appropriate imaging modality to identify the location of LPN lesions [7]. In our report, no obvious amyotrophy of the pectoralis major muscle was detected with ultrasonography, nor any space-occupying lesion in the nerve course or focal enlargement of the nerve. The ultrasound guide perfected our procedures, as well as the sensitive and motor test previous to the pRF, making sure it was precise and with a minimum damage of the structures in the vicinity.

pRF was considered as a therapeutic approach for this clinical case because it offers the unique advantages of pain relief without significant damage to nervous tissue or painful sequelae like continuous radiofrequency does. It is especially useful in cases of neuropathic pain, in sensitive-motor nerves, or pure sensitive nerves with a large cutaneous expression. The mechanism by which pRF controls pain is still unclear, but it may involve a temperature-independent pathway mediated by a rapidly changing electric and magnetic field. Some studies suggest that the rapidly changing electrical fields generated by pRF can affect neuronal membranes, changing synaptic signaling and causing electroporation [8]. It is thought to alter the transmission of pain signals via a pathway involving c-Fos (increasing its expression), and activate transcription factor 3 (ATF3, an indicator of “cellular stress”) in small-diameter C and Aδ fibers [4,9].

Some articles report that physiotherapy including an exercise program and changes in work activities may help to prevent further damage [2,7]. In our report, our patient did a cycle of physiotherapy that didn’t help in her intense pain. By approaching and controlling her pain, we improved not only her quality of life and functionality but also allowed a window to perform the modifications and strength program without any pain.

## Conclusions

Though extremely rare, isolated injuries of the LPN should be considered in the differential diagnosis of a refractory shoulder and chest pain, when the medical exam is inconclusive, including neurological examination and imaging. The diagnostic and therapeutic block test with anesthetics was frankly positive in this case, and so was the pRF procedure.

This case report displays the importance of proper clinical judgment and reasoning that, in combination with the know-how of a specific pain management technique like pRF, led to the resolution of our patient's symptoms and functional repercussions.
